# PEG-Protein Interaction Induced Contraction of NalD Chains

**DOI:** 10.1371/journal.pone.0096616

**Published:** 2014-05-08

**Authors:** Jiyan Yu, Weizhong Chen, Chi Wu, Hao Chen

**Affiliations:** 1 Department of Chemical Physics, University of Science and Technology of China, Hefei, China; 2 Coordination Chemistry Institute and the State Key Laboratory of Coordination Chemistry, School of Chemistry and Chemical Engineering, Nanjing University, Nanjing, China; 3 Department of Chemistry, The Chinese University of Hong Kong, Hong Kong, China; University of South Carolina, United States of America

## Abstract

In a recent attempt to crystallize a regulator of MexAB-OprM multi-drug efflux systems in *Pseudomonas aeruginosa* (NalD), we found that adding polyethylene glycol (PEG3350, Mw = 3,350 g/mol) into the protein solution increases the speed of NalD migration in gel electrophoresis, signaling a smaller hydrodynamic size. At first we conjectured that NalD was degraded unexpectedly by PEG; however, we found that there was no change in its molar mass by MALDI-TOF characterization. Moreover, we found that adding polyacrylic acid (PAA) into the solution mixture returned the NalD migration to its normal speed. Furthermore, our analytic ultracentrifugation and dynamic laser light scattering results directly reveal that NalD interacts with PEG so that individual NalD chains gradually shrink as more PEG chains are added in the range of 10–50 mg/mL. Size exclusion chromatography also confirms that the NalD chain shrinks in the presence of PEG. A combination of these results indicates that PEG3350 chains can complex with NalD to induce an intra-protein chain contraction, presumably via the formation of hydrogen bond between –C-O-C– on PEG and –COOH on NalD, resulting in a smaller hydrodynamic size (faster migration) and a higher apparent molar mass. Note that because the presence of PEG affects osmotic pressure, it is considered to be a precipitator of protein crystallization. Our current finding reveals that the interaction of PEG/protein may play a significant role in protein crystallization. The complexation potentially makes the protein chain segments less flexible, and consequently makes crystallization easier. Hopefully, our current results will stimulate further studies in this direction.

## Introduction

Among the variety of substances which are used to induce protein crystallization, polyethylene glycol (PEG, short chains of polyethylene oxide) is one of the most widely used and necessary ingredients for protein crystallization [1]–[7]. A rough estimate shows that PEG has been used for more than 70% of all successfully crystallized proteins [8]. A frequently given qualitative explanation for PEG induced protein crystallization is that it has less of an effect on the electrostatic repulsion of protein chains because it is a neutral surfactant so that its addition only affects osmotic pressure and generates an attractive depletion force among protein chains. Other interactions are also found between PEG and proteins, including the formation of hydrogen bonds between the oxygens in the –C-O-C– group of PEG and the carboxyl and phenolic -OH groups of protein [9]–[11]. To our knowledge, the mechanism of how exactly PEG chains promote protein crystallization remains obscure at the molecular level. As such, manipulating protein crystallization is still very much a trial-and-error process.

Recently, we attempted to crystallize NalD, a protein of the TetR family of 212 amino acids, in *Pseudomonas aeruginosa* PAO1, which represses the MexAB-OprM multi-drug efflux operon [12], [13]. Surprisingly, we found that the NalD band moved faster in SDS-PAGE when PEG with a molar mass of 3,350 g/mol was added in the solution mixture. Initially, we conjectured that NalD was unexpectedly degraded by PEG but we were unable to figure out in what way PEG is catalytic. Finally, we realized that there exists some special interaction between NalD and PEG. Previously, Onuma et al. [8] found that TAA protein chains also interact with PEG and speculated that the interaction led to the formation of a network-like structure because of the possible entanglement of PEG chains in the solution mixture. However, such a speculation cannot explain a smaller size of NalD in the presence of PEG. Namely, NalD migration in SDS-PAGE speeded up even with the addition of a small amount of PEG3350 (10 mg/mL) wherein there was no entanglement of PEG chains. On the other hand, if the PEG chains entangled with each other, the NalD migration in SDS-PAGE would be slower rather than faster.

To understand this abnormal phenomenon, we used a combination of matrix-assisted laser desorption/ionization time of flight (MALDI-TOF) mass spectrometry, dynamic laser light scattering (LLS), size exclusion chromatography (SEC), and analytic ultracentrifuge to reveal why adding PEG into the solution mixture makes NalD migrate faster in SDS-PAGE. We found that the interaction between NalD and PEG, presumably the formation of hydrogen bond between –C-O-C– in PEG and –COOH and HO-Ar- in NalD, led to the intrachain contraction of individual NalD chains when the PEG concentration was low, namely a smaller hydrodynamic size. Indirectly, we showed that by adding polyacrylic acid (PAA) with many –COOH groups, a hydrogen bond forming competitor of NalD, NalD migration returned to normal in SDS-PAGE despite the presence of PEG.

## Materials and Methods

### Construction, Expression, and Purification of NalD

The *nalD* gene was amplified from *Pseudomonas aeruginosa* PAO1 by PCR and was cloned into a PET-30a vector between the NdeI and XhoI sites. The NalD-PET-30a plasmid was transformed into a BL21 star (DE3) strain. After growing overnight, 10 mL of the culture from a single colony was diluted into a 1.0-L autoclaved LB medium that contained 30-mg kanamycin to enable bacteria to grow at 37°C, 250 rpm until OD_600_ reached 0.6. The expression of NalD was achieved by further addition of IPTG with a final concentration of 0.5 mM and further incubation for 4 h at 30°C. The cells were harvested at room temperature and stored at −80°C for subsequent uses.

A 1.0-L cell pellet was re-suspended and lysed in 15-mL buffer (10-mM Tris-HCl, pH = 6.8, 50-mM NaCl, 5-mM 2-mercaptoethanol (β-ME), and 70-U Takara DNaseI) by sonication and then centrifuged down at 12,000 rpm for 25 min. The resulting supernatant was filtered through a 0.45-µm filter and loaded to an SFF column equilibrated with buffer A (10-mM Tris-HCl, pH = 6.8 and 5-mM β-ME). Such preliminarily purified NalD was eluted by 45% of buffer B (10-mM Tris-HCl, pH = 6.8, 5-mM β-ME, and 1-M NaCl). The sample was then loaded to a Heparin column and different fractions were collected by 40% of buffer D (10-mM Tris-HCl, pH = 8.0, 2-mM DTT, and 1.0-M NaCl). Finally, the mixed samples were loaded to a Ni-NTA column equilibrated with buffer E (10-mM Tris-HCl, pH = 8.0, 5-mM β-ME, and 100-mM NaCl) and the fraction was pooled by 20% of buffer F (10-mM Tris-HCl, pH = 8.0, 5-mM β-ME, 100-mM NaCl, and 500-mM imidazole). The pure NalD protein was eluted and purified in 18% of buffer D by a monoQ column. The NalD protein was harvested by a Millipore concentration tube and its purity was verified by 14% SDS-PAGE gel.

### Dynamic Laser Light Scattering (LLS)

The PEG3350 (M = 3,350 g/mol, purchased from Sigma) solution was prepared by dissolving solid PEG3350 in a proper amount of Milli-Q water. The purified NalD protein (1.0 mg/mL) and PEG3350 (400 mg/mL) solutions were clarified by 20-nm (Whatman, Anotop 10) and 0.45-µm hydrophilic PTFE (Millipore) filters, respectively, just before mixing them for LLS measurements. The solution mixtures of NalD and PEG (C_PEG_ = 0–10 mg/mL) were prepared by adding a proper amount of the clarified PEG solution and Milli-Q water into a fixed amount of the clarified NalD solution. Each solution mixture was measured by dynamic LLS (ALV/DLS/SLS-5022F) at a scattering angle of 90° at 20°C. Using only one angle is because the scattering intensity is nearly angular independent. The theory and other details of LLS instrumentation can be found elsewhere [14].

### Analytical Ultracentrifuge

An analytical ultracentrifuge (Beckman Optima XL-I with a UV detection light of 280 nm and a centrifuge speed of 45,000 rpm) was used to measure the sedimentation coefficient and molar mass of NalD in aqueous solutions without and with different amounts of PEG3350 in the range of 10–75 mg/mL at 20°C. The apparent weight average molar masses of NalD were estimated from the peak positions.

### Viscosity Measurements

The viscosity of each solution mixture was measured by using an Ubbelohde viscometer at 20°C, which is required in the calculation of the hydrodynamic radius distribution in dynamic LLS and the apparent weight average molar mass of NalD in the ultracentrifugation. The time required for each solution mixture to pass the two marks on the capillary tube was measured three times and the error bar was less than ±0.2 s. The solvent viscosities (Milli-Q water) at different temperatures were also measured the same way.

### SDS-PAGE Analysis

SDS-PAGE (14%) was used to investigate the interaction between NalD and PEG in the solution mixture of NalD and PEG with different PEG concentrations (10–150 mg/mL). We also added different amounts of polyacrylic acid (PAA) into the solution mixture of NalD and PEG.

### Size Exclusion Chromatography

FPLC system (ÄKTA) equipped with Superdex 200 10/300 GL (GE healthcare) was used. 200 uL NalD protein solution was injected into FPLC system and eluted with GF buffer (10-mM Tris-HCl, pH = 8.0, 5-mM β-ME, 300-mM NaCl). The analysis was executed at 4°C and the detection wavelength was set at 280 nm. For the mixture of NalD and PEG or PAA, 5 mg/mL PEG or 3 mg/mL PAA was mixed with the NalD solution and GF buffer.

## Results and Discussions

**Figure 1:**
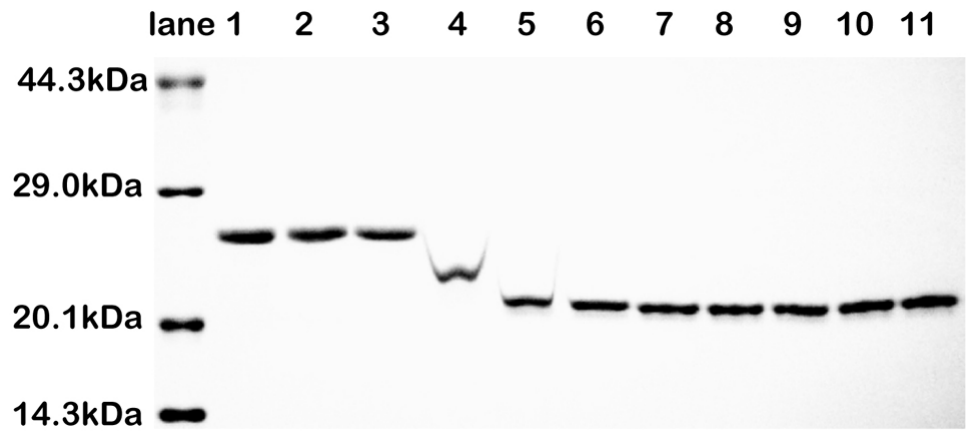
Migration of NalD protein in 14% SDS-PAGE with different amounts of PEG3350. Lane 1 is pure NalD protein; and Lanes 2∼11 correspond to different amounts of added PEG3350 (1, 2, 5, 10, 20, 30, 50, 75, 100, and 150 mg/mL).

Figure 1 shows that the NalD protein chains migrate faster in SDS-PAGE after the addition of PEG3350. In other words, the NalD protein band moves to a position with a lower molar mass as more PEG3350 chains are added from 0 to 10 mg/mL. The movement essentially stops when the PEG3350 concentration reaches 10 mg/mL. Initially, we thought that NalD was unexpectedly degraded into smaller fragments in the presence of PEG3350 because of their faster migration rate. However, we were unable to figure out the reason behind the unexpected catalytic activity, or NalD ‘chopping’, of PEG. In addition, it is also mysterious how each NalD chain is chopped into small similar sized fragments given that the faster moving NalD band is fairly narrow. This problem perplexed us for a few months.

We later realized that a previous study showed that synthetic grafted copolymer of poly(acrylic acid)-g-poly(ethylene glycol) (PAA-g-PEG) could form large aggregates via the hydrogen bonding between the carboxyl groups on PAA and the ether oxygen on the PEG [15]–[17]. Further, a literature search revealed other relevant results such as the formation of water-soluble complexes by pepsin and human serum albumin (HSA) with PEG via the hydrogen bonding between the ether groups in PEG and the carboxyl or the phenolic OH groups in the proteins [9]–[11]. This type of hydrogen bonding induced complexation then led us to conjecture that the smaller hydrodynamic size and a faster migration in SDS-PAGE might be due to the complexation between each NalD chain and some PEG chains which results in the intrachain contraction of individual NalD chains because NalD has many carboxyl groups (11 Asp residues and 21 Glu residues).

**Figure 2:**
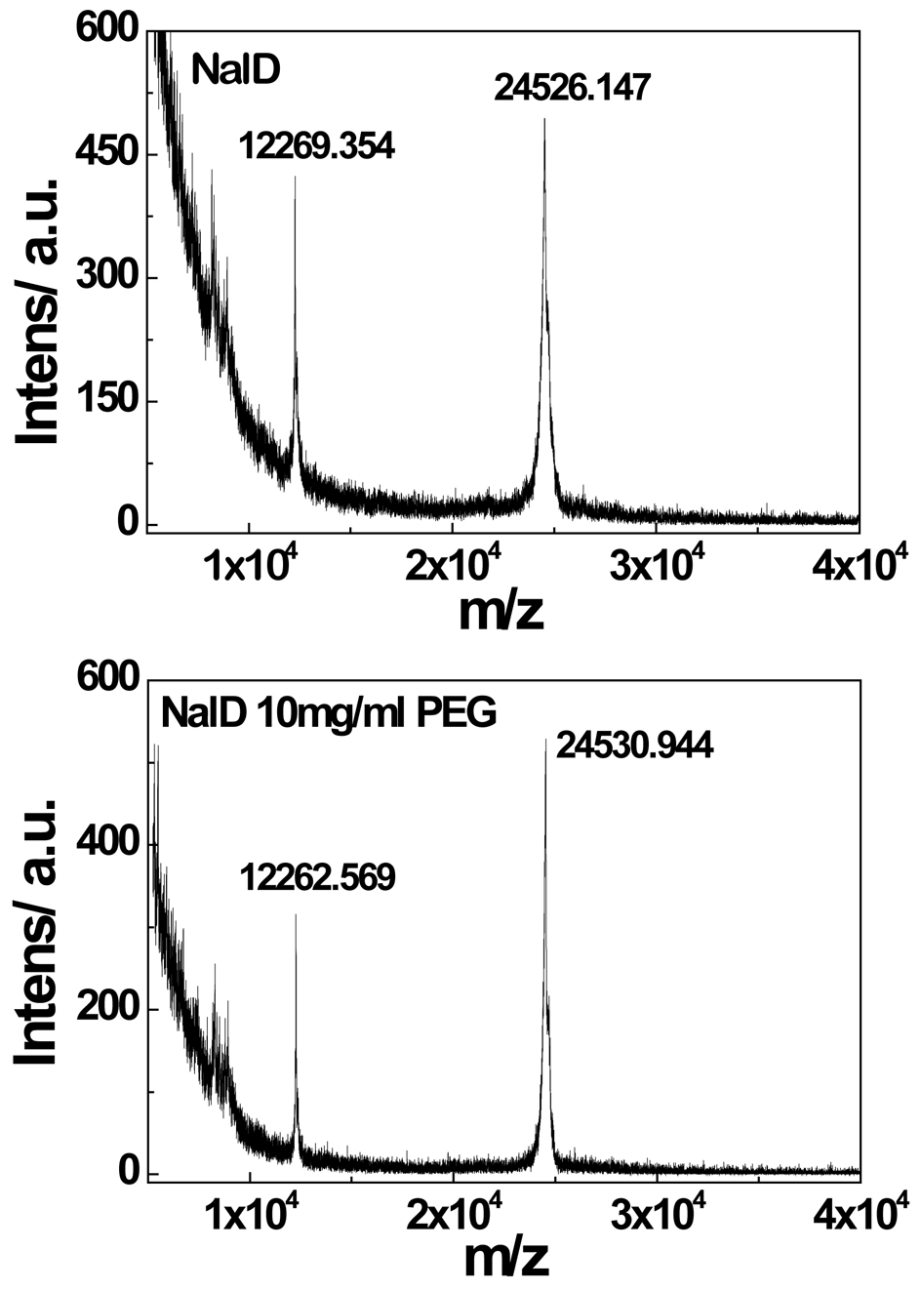
MALDI-TOF characterization of molar mass of NalD protein without and with PEG3350. The peaks at ∼24520 and 12260 represent NalD protein chains with one and two protons, respectively; the matrix is sinapic acid and a linear mode was used for chains with a molar mass higher than 6000.

**Figure 3:**
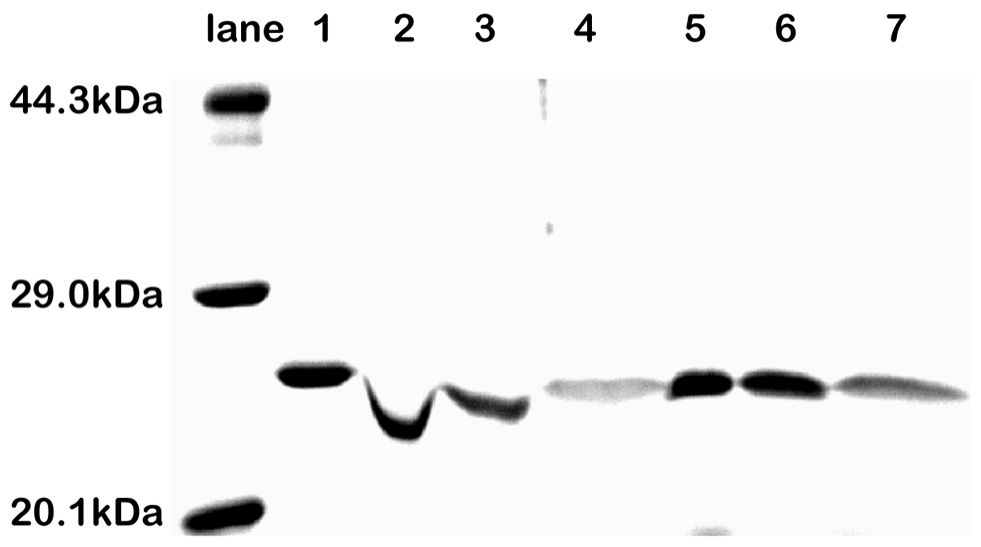
Migration of NalD protein in 14% SDS-PAGE with PEG3350 and PAA. Lanes 1 and 5 are pure NalD; Lanes 2–4, NalD solutions with PEG3350 (3 mg/mL) and different amounts of PAA (0, 20, and 50 mg/mL, respectively); Lanes 6 and 7, NalD with PAA (20 and 50 mg/mL, respectively) but no PEG.

To confirm such a hypothesis, we used MALDI-TOF mass spectrometry to characterize the molar mass of NalD before and after it was mixed with PEG3350 and found that there was indeed no change in its molar mass, as shown in Figure 2. Therefore, we ruled out the degradation possibility. Next, we added PAA (M_w_ ∼ 3,000 g/mol), a hydrogen bonding competitor because it has ∼30–COOH groups, in the solution mixture of NalD and PEG to determine whether it could replace NalD in the complexes. As expected, the addition of PAA resets the NalD migration back to normal in SDS-PAGE, as shown in Figure 3, even in the presence of PEG in comparison with the NalD band in Lane 2 without PAA. Figure 3 also shows that in the pure solution of NalD, adding PAA has no effect on the NalD migration (Lanes 6 and 7). A combination of the MALDI-TOF and the PAA replacement results confirms our hypothesis.

**Figure 4:**
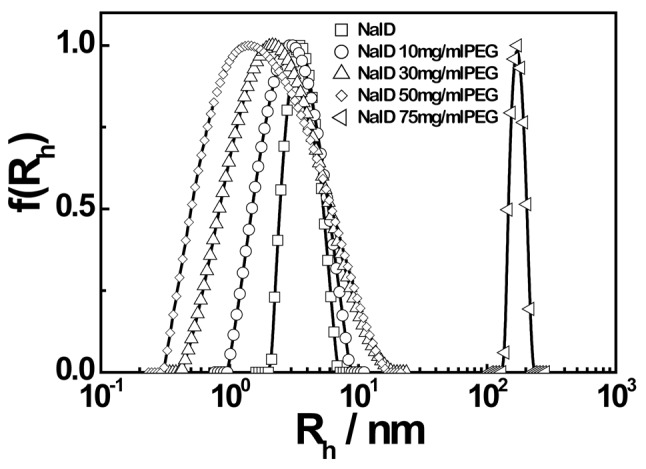
Scattering intensity weighted hydrodynamic radius distributions of NalD chains in presence of different amounts of PEG3350.

Note that in SDS-PAGE, 1 mg/mL of anionic surfactant, SDS, is added. The alkyl tail of SDS interacts with the hydrophobic part of protein chains while its hydrophilic anionic sulfonic head stays on the periphery to make each protein chains negatively charged so that the interaction between NalD and PEG would be weaker. To directly confirm this complexation induced intrachain contraction in a mimic native condition, we use dynamic laser light scattering (LLS) to directly measure the hydrodynamic radius distribution of NalD in aqueous solutions without and with different amounts of PEG3350, as shown in Figure 4. When more PEG is added, the hydrodynamic radius distribution shifts toward the direction of smaller hydrodynamic radius and becomes broader, clearly indicating the intrachain contraction of NalD. Table 1 summarizes the average hydrodynamic radius (<R_h_>) calculated from each corresponding hydrodynamic radius distribution in Figure 4, where the viscosity of each solution measured by an Ubbelohde viscometer was used in the calculation.

**Table 1:** Solution viscosity and average hydrodynamic radius (<R_h_>) of NalD in aqueous solutions with different amounts of PEG3350.

PEG3350/(mg/mL)	0	10	30	50
Viscosity/cp	1.01	1.14	1.46	1.80
<*R_h_*>/nm	3.80	3.09	2.46	1.87

Note that PEG chains can complex with NalD inside to induce the intrachain contraction or with two or more NalD chains to “bridge” them together to form large interchain aggregates. A competition between these two processes always exists. When the PEG concentration is low, the intrachain process is dominant so that the peak in Figure 4 shifts to the left while the interchain process is only reflected in the peak broadening. Figure 4 also shows that when the PEG concentration is higher than 75 mg/mL, the interchain association over-shadows the intrachain contraction so that the peak suddenly shifts to the region of 100–200 nm, indicating the formation of large interchain NalD/PEG aggregates. It should be noted that no large aggregates were detected in SDS-PAGE even when the PEG concentration was as high as 150 mg/mL. Presumably, this is due to the presence of the surface active agent SDS that stabilizes NalD and weakens the hydrogen bonding between NalD and PEG.

**Figure 5:**
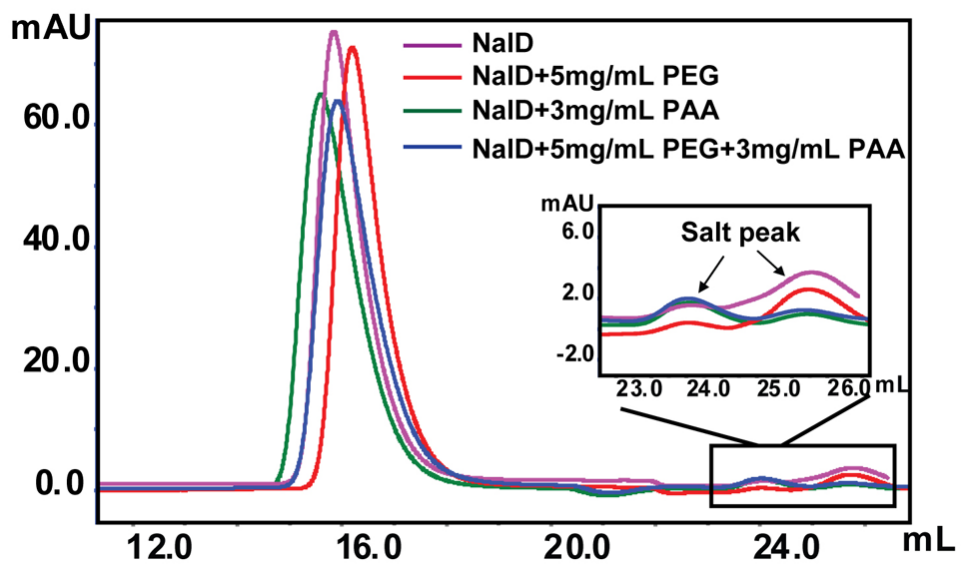
Size exclusion chromatography analysis of NalD protein with PEG and PAA.

Size exclusion chromatography (SEC) is a method that separates macromolecules by their different sizes; large macromolecules are excluded from small pores inside beads packed inside the column so that they are eluted out rapidly, while small macromolecules eluted out later. Figure 5 shows that in the presence of PEG3350 (5 mg/mL), the NalD chains are eluted out later, also indicating the contraction of individual NalD chains. The addition of PAA (3 mg/mL) into the mixture of NalD and PEG returns the NalD elution back to normal, which confirms that anionic surfactant PAA as a hydrogen bonding competitor that can replace NalD in the NalD/PEG complexes. In addition, this supports our hypothesis that it is the hydrogen bonding induced intrachain complexation between NalD and PEG that leads to the chain contraction of NalD and a smaller hydrodynamic size. Note that adding PAA (3 mg/mL) actually makes NalD eluting faster, presumably because the interaction of PAA and NalD makes its hydrodynamic size larger. The complexation between proteins and PAA was also previously studied by using turbidimetry and isothermal calorimetric titrations [18]. On the other hand, our SDS-PAGE result shows that the addition of PAA has no effect on the NalD migration. More PAA was needed to return the NalD migration back to normal in comparison with size exclusion chromatography because of the presence of anionic SDS.

**Figure 6:**
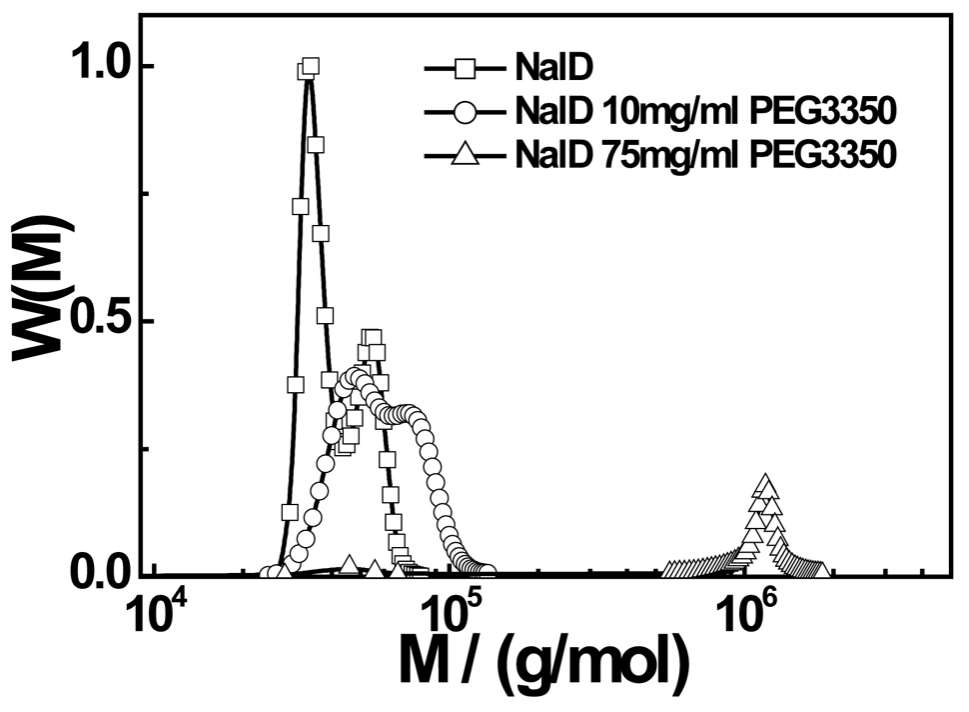
Molar mass distributions of NalD from ultracentrifugation analysis in presence of different amounts of PEG3350.

In order to directly reveal the complexation between NalD and PEG, we used an analytical ultracentrifuge to measure the molar mass distribution of NalD in the presence of different amounts of PEG3350, as shown in Figure 6. Before the addition of PEG3350, the molar mass distribution already has two peaks, reflecting that some NalD chains in the solution exist as dimmers and oligomers. The addition of PEG3350 (10 mg/mL) shifts the two peaks to the higher molar masses, clearly signaling the complexation because PEG itself has a much lower molar mass. Further increase of the PEG3350 concentration to 75 mg/mL finally diminishes the peak related to the intrachain complexation and results in a peak located in the range of ∼10^6^ g/mol, i.e. the formation of large interchain aggregates, agreeing well with our LLS results. The quantitative results from Figure 5 are summarized in Table 2.

**Table 2:** Ultracentrifugation characterization of apparent molar masses of NalD (peak values) without and with different amounts of PEG3350.

PEG3350	M_peak 1_/(g/mol)	M_peak 2_/(g/mol)
0 mg/mL	3.40×10^4^	5.38×10^4^
10 mg/mL	4.77×10^4^	7.35×10^4^
75 mg/mL	1.18×10^6^	

**Figure 7:**
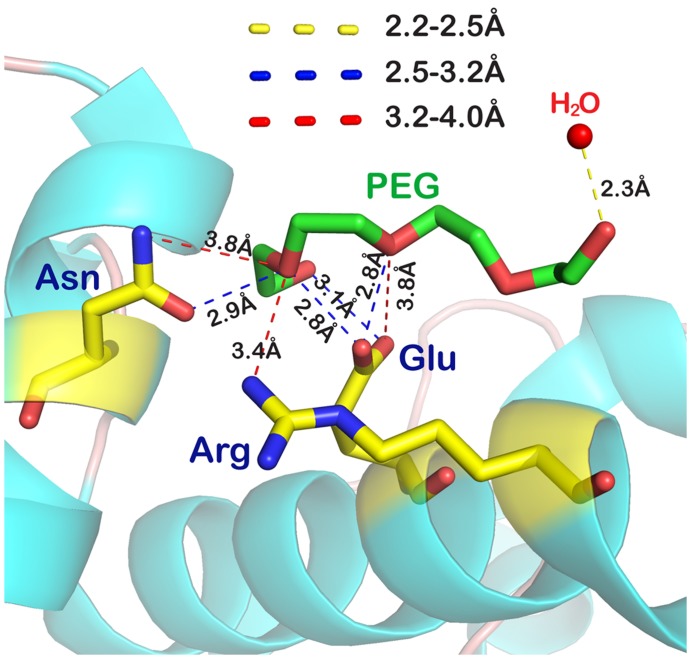
Part structure of TetR that shows interaction between PEG and TetR. The distances between –C-O-C- on PEG and –COOH and –NH_2_ on TetR and H_2_O are labeled.

After confirming the complexation between NalD and PEG and its effect on the protein purification, we have to consider its implications in other aspects. A search of the protein data bank reveals many direct instances of the PEG-protein interaction embedded in protein structures. The crystal structure of TetR protein (PDB ID 3ZQI), a homologous of NalD, has small PEG segments around protein [19], indicating that some PEG fragments must firmly bind to protein because those flexible fragments are invisible in the electron density map and omitted from the structure analysis. Figure 7 shows a part of the TetR structure wherein the hydrogen bonds between –C-O-C- on PEG and amino acid residues on TetR (–COOH of Glu, –NH_2_ of Asn and –NH– of Arg) are clear. Jeffrey categorizes hydrogen bonds with a donor-acceptor distance in the ranges of 2.2–2.5, 2.5–3.2 and 3.2–4.0 Å as “strong, mostly covalent”, “moderate, mostly electrostatic”, and “weak, electrostatic”, respectively [20]. As shown in Figure 7, the hydrogen bonds between PEG and TetR are in the range 2.8–3.8 Å, confirming that the NalD/PEG interaction indeed exists. As a matter of fact, such PEG segments exist in hundreds of protein structures, suggesting that the protein/PEG complexation is a general phenomenon and plays an important role in the crystallization of some proteins.

## Conclusion

As a widely used and efficient agent, PEG plays an important role in protein crystallization. In the current study, by using a combination of MALDI-TOF mass spectrometry, laser light scattering, analytic ultracentrifuge, and size exclusion chromatography, we have revealed that the fast migration of the NalD band in SDS-PAGE in the presence of PEG is related to the intrachain contraction of NalD (the shrinkage of NalD with a smaller hydrodynamic size) that is induced by the hydrogen bonding between individual NalD chains and PEG via the carboxylic groups in NalD and the ether groups in PEG. Our results agree well with the widespread existence of PEG segments in some resolved protein crystal structures. Such a confirmed intrachain complexation has some profound implications in protein crystallization. To name a few, 1) there is hydrogen bonding between protein and PEG, especially when protein contains a large amount of –COOH and -Ar-OH groups; 2) the concentration and length of PEG should be properly tuned so that the interchain complexation is avoided; 3) one might be able to use the intrachain complexation to make the chain segments of a protein less flexible to facilitate the packing and crystallization of protein chains because each PEG chain acts like a “cross-linking” agent inside individual protein chains; 4) some PEG binding sites near the catalytic core in protein crystals might be used as potential targets of small molecules to adjust the protein activity in the drug and inhibitor designs; and 5) one can purposely design some specific hydrogen bonding oligomers or short polymer chains to facilitate the crystallization of a given protein.
